# The impact of the COVID-19 pandemic on unilateral inguinal hernioplasty surgery in Brazil

**DOI:** 10.1590/0100-6991e-20223316-en

**Published:** 2022-09-21

**Authors:** GIOVANA SALVIANO BRAGA GARCIA, KARINE COELHO DA SILVA FERREIRA, LIGIA SOUZA WANDERLEY, JULIA MARIA MENDONÇA MACHADO PINHEIRO, ISADORA MACIEL KORSACK, KATIA GLEICIELLY FRIGOTTO

**Affiliations:** 1 - Universidade do Grande Rio Professor José Herdy, - Rio de Janeiro - RJ - Brasil; 2 - Universidade Federal do Estado do Rio de Janeiro, - Rio de Janeiro - RJ - Brasil

**Keywords:** Inguinal Hernia, COVID-19, Herniorrhaphy, Hérnia Inguinal, COVID-19, Herniorrafia

## Abstract

**Objective:**

to analyze data from patients hospitalized for unilateral inguinal hernioplasty in Brazil in the year before the COVID-19 pandemic, and during the period of the pandemic.

**Methods:**

this is a descriptive study, using data referring to hospitalizations for the surgical procedure of unilateral inguinal hernioplasty in Brazil from March 2019 to February 2020, comparing with data from March 2020 to February 2021. Data were collected from the Hospital Information System (SIH/SUS) and the selected variables were: number of hospitalizations, average hospital stay rate and mortality rate.

**Results:**

in all, 119,312 hospitalizations were performed for unilateral inguinal hernioplasty in Brazil from March 2019 to February 2020. During the pandemic period, 53,445 hospitalizations were recorded for this procedure. The average hospital stay increased compared to the previous year. The mortality rate recorded in the year before the pandemic was 0.11, while in the period of the pandemic, it was 0.20.

**Conclusion:**

It was observed that during the period of the COVID-19 pandemic in Brazil, the number of hospitalizations for unilateral inguinal hernioplasty was reduced by 55,21%. However, there was a significant increase in the mortality rate of this procedure. These results can be explained by the increase in mortality in patients infected with the SARS-CoV-2 virus, and also by the restriction of performing elective surgeries, prioritizing emergency situations, which are more complicated, and consequently, with higher mortality.

## RESUMO

**Objetivo:**

analisar os dados dos pacientes internados para realização de hernioplastia inguinal unilateral no Brasil no ano anterior à pandemia de COVID-19, e durante o período da pandemia no país.

**Métodos:**

trata-se de um estudo descritivo, utilizando dados referentes às internações para realização do procedimento cirúrgico de hernioplastia inguinal unilateral no Brasil no período de março de 2019 a fevereiro de 2020, comparando com os dados de março de 2020 até fevereiro de 2021. Os dados foram coletados do Sistema de Informações Hospitalares (SIH/SUS) e as variáveis selecionadas foram: número de internações, taxa média de permanência hospitalar e taxa de mortalidade.

**Resultados:**

ao todo, foram realizadas 119.312 internações para realização de hernioplastia inguinal unilateral no Brasil no período de março de 2019 a fevereiro de 2020. Já durante o período de pandemia no país, foram registradas 53.445 internações para este procedimento. A média de permanência hospitalar aumentou em relação ao ano anterior. A taxa de mortalidade registrada no ano anterior à pandemia foi de 0,11, já no período da pandemia, foi de 0,20.

**Conclusão:**

foi observado que durante o período de pico da pandemia de COVID-19 no Brasil, o número de internações para realização de hernioplastia inguinal unilateral foi reduzido em 55,21%. Contudo, houve um aumento significativo da taxa de mortalidade desse procedimento. Esse resultado pode ser explicado pelo aumento da mortalidade em pacientes infectados pelo vírus SARS-CoV-2, e também pela restrição da realização de procedimentos cirúrgicos eletivos nesse período, priorizando quadros emergenciais, os quais são mais complicados, e consequentemente, com maior mortalidade.

## INTRODUCTION

Hernia is of Latin origin and means rupture^1^ ^-^ ^2^. It is defined as an abnormal protrusion of a tissue or organ due to a fragility in its surrounding walls^2^ ^-^ ^3^. Specifically, inguinal hernia consists of the protrusion of an intestinal loop or, more rarely, of another intra-abdominal viscera, through an orifice in the abdominal wall in the groin region, or through the internal inguinal ring^4^. Genetic predisposition, related to situations of increased pressure in the abdominal cavity, favors the emergence and increase of such hernias^4^. It is important to emphasize that this pathology is one of the most frequent in general surgery^5^ and it is erroneously considered by many to be a minor complication. However, it has an important impact on the patient’s social life^4^.

Inguinal hernia is the most frequent among all hernias, accounting for approximately 75% of abdominal wall hernias^2^ ^,^ ^4^. Inguinal hernias can be divided into two types: direct, which form directly at a point on the weakened abdominal wall; and indirect, which is formed by the passage of the intestinal loop through the internal inguinal ring^2^. Inguinal hernias are commonly associated with men, since their risk of development in males is around 25%, while in females it is only 5%^4^.

From a clinical point of view, they vary from asymptomatic to severe conditions, which can result in abdominal sepsis and/or peritonitis. The predominant symptom is localized pain resulting from dilation of the hernia ring by the hernia content^4^. The classic clinical presentation is composed of unilateral or bilateral bulging of the inguinal region, whether cause by effort or spontaneously, and the presence or absence of pain at the site^4^. In men, there may be bulging in the scrotal region^4^. Complex conditions include repeated vomiting, abdominal distension, diffuse abdominal pain, hyperemia in the inguinal or scrotal region, cessation of gas and feces elimination, fever, and poor general condition^4^.

The diagnosis is mostly clinical, anamnesis and physical examination being essential. The two together have a sensitivity of 74.5% and a specificity of 96%^5^. Imaging tests, such as ultrasound, can be performed in case of doubt, or in the presence of differential diagnoses, such as lymph node enlargement, hydrocele, pubeitis, osteitis, among others^5^. After diagnosis, the most indicated conduct is hernioplasty, ideally in an elective manner, in cases where manual reduction of the hernia is possible^4^. Symptomatic patients, with pain on exertion, prostatism, chronic constipation, and classified as ASA I or II (American Society of Anesthesiology) constitute the group that will benefit most from early hernioplasty^5^.

Complications associated with inguinal hernioplasty are rare^2^. Every surgical procedure has risks, however. Pain, seroma, hematoma, and hernia recurrence are the most prominent complications of this procedure^2^ ^,^ ^6^. On the other hand, postponing the operation may entail the risk of strangulation of visceral organs, with additional risks of gangrene, perforation, and infection of the peritoneal cavity^6^. As a result, few cases are left without surgery and are submitted to observational management, the risk of incarceration being greater soon after clinical manifestations^4^.

Due to the SARS-CoV-2 virus pandemic, most health services and hospitals were congested at the height of the pandemic due to the treatment of patients with COVID-19, restricting surgical procedures to emergency only. This occurred because there was a need to release more beds and greater availability of mechanical ventilator in case of increased demand for them. In addition, the pause in elective surgery aimed at preventing contamination of patients undergoing procedures with low morbidity and mortality^7^.

Thus, hernia repairs, which are only performed in the context of an emergency when it is not possible to manually reduce the hernia content^4^, were rescheduled for a more opportune moment in the pandemic. However, the delay in performing an elective surgery can bring serious risks to the patient^7^ ^-^ ^9^, which can lead to possible complications of the disease^9^. This makes surgery a more complex and risky procedure, such as, for example, emergency surgery for incarcerated inguinal hernia, with higher morbidity and mortality when compared with the elective approach without incarceration^6^ ^,^ ^10^. Furthermore, even being a low morbidity and mortality operation, hernioplasty can be fatal in patients infected with COVID-19^11^.

Thus, in view of the current pandemic context, this study aims to analyze the impact of the COVID-19 pandemic on unilateral inguinal hernioplasty surgery, through data from patients hospitalized for this procedure in Brazil in the year before the pandemic, and during the peak pandemic period in the country.

## METHODS

This is a descriptive study, using data referring to hospitalizations for unilateral inguinal hernioplasty in Brazil from March 2019 to February 2020, before the COVID-19 pandemic arrived in the country, and from March 2020 to February 2021, during the peak period of the COVID-19 pandemic in Brazil.

We collected data from the Hospital Information System (SIH/SUS) and the selected variables were number of admissions, average hospital stay rate, and mortality rate per 100 admissions. We accessed the information in the option “Access to information”, followed by the item “Health information (TABNET)” and “Hospital Production (SIH/SUS)”. Then, we selected the procedure “04.07.04.010-2 - Inguinal/crural hernioplasty (unilateral)”, and the time period studied, first between March 2019 and February 2020, and then from March 2020 to February 2021.

We used the Microsoft Excel software for tabulation, data analysis, and graphical representation.

## RESULTS

**Figure 1:**
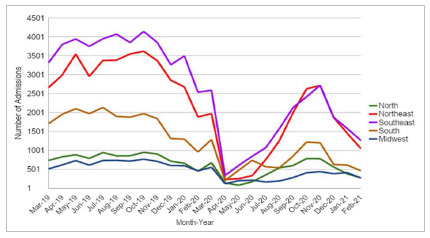
Number of admissions for unilateral inguinal hernioplasty in Brazil from March 2019 to February 2021.

There were 119,312 admissions for unilateral inguinal hernioplasty in Brazil from March 2019 to February 2020, in which the Southeast Region recorded the highest number, 43,961 admissions (36.85%), followed by the Northeast (30.90%), South (17.64%), North (8.05%), and Midwest (6.56%). During the pandemic period in the country, from March 2020 to February 2021, 53,445 hospitalizations were registered for the same procedure, resulting in a reduction of 55.21% compared with the previous period, with the Southeast Region responsible for 35.60% of hospitalizations, followed by the Northeast (30.94%), South Region (16.50%), North (10.07%), and Midwest (6.89%) (Figure 1).

**Figure 2:**
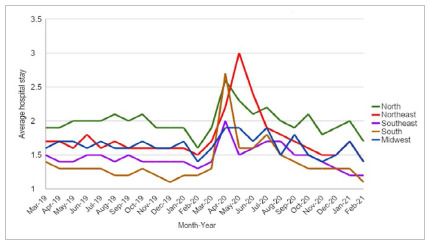
Average hospital stay of unilateral inguinal hernioplasty surgery in Brazil from March 2019 to February 2021.

The mean hospital stay increased when compared with the previous year. During the period before the pandemic, it averaged 1.58, and during the pandemic period, 1.72 (Figure 2).

The mortality rate recorded in the year before the pandemic was 0.11 (Table 1), while in the period of the COVID-19 pandemic, there was an increase in mortality, to 0.20 (Table 2). The largest increase in mortality took place in the Midwest Region, from 0.07 before the pandemic to 0.27during it, an increase of 285.71%. In the Northeast region, the mortality rate went from 0.12 to 0.36 (200% increase), in the Southeast, from 0.10 to 0.29 (190.0% increase), in the North, from 0.10 to 0.13 (30.0% increase), and in the South, the mortality rate remained very close to the previous year, around 0.12.

**Table 1:** Mortality rate of Unilateral Inguinal Hernioplasty in each Region from March 2019 to February 2020.

Region	2019/Mar	2019/Apr	2019/May	2019/Jun	2019/Jul	2019/Aug	2019/Sep	2019/Oct	2019/Nov	2019/Dec	2020/Jan	2020/ Feb	Total
North	-	-	0.34	-	0.11	0.12	0.12	0.21	0.11	-	0.15	-	0.10
North East	0.11	0.17	0.06	0.20	0.12	0.06	0.20	0.19	0.06	0.21	0.04	0.05	0.12
Southeast	0.09	-	0.08	0.11	0.15	0.12	0.05	0.12	0.16	0.06	0.14	0.12	0.10
South	0.23	-	0.14	0.15	0.05	0.21	0.16	0.15	0.11	0.08	0.08	0.10	0.12
Midwest	-	-	0.14	-	0.14	0.27	-	0.13	0.14	-	-	-	0.07
Brazil	0.11	0.05	0.11	0.13	0.12	0.13	0.12	0.16	0.11	0.10	0.09	0.08	0.11

**Table 2:** Mortality rate of Unilateral Inguinal Hernioplasty in each Region from March 2020 to February 2021.

Region	2020/Mar	2020/Apr	2020/May	2020/Jun	2020/Jul	2020/Aug	2020/Sep	2020/Oct	2020/Nov	2020/Dec	2021/Jan	2021/Feb	Total
North	0.44	-	-	0.57	-	-	0.17	0.25	0.13	-	-	-	0.13
North East	0.10	1.27	0.39	0.88	0.26	0.40	0.15	0.23	0.11	0.05	0.07	0.38	0.36
Southeast	0.19	0.58	0.66	0.47	0.28	0.25	0.19	0.12	0.22	0.16	0.25	0.16	0.29
South	0.08	-	-	-	0.18	-	0.12	0.08	0.17	0.48	0.33	-	0.12
Midwest	-	-	0.49	-	0.59	1.03	0.35	0.24	-	0.52	-	-	0.27
Brazil	0.16	0.47	0.37	0.34	0.24	0.27	0.17	0.17	0.15	0.17	0.16	0.18	0.20

## DISCUSSION

Due to the COVID-19 pandemic, adaptations to the functioning of the health system around the world were necessary to face the challenge of treating many critically ill patients with a new disease, while keeping essential health services functioning properly^7^.

As the pandemic progressed, elective procedures were temporarily interrupted to reduce the risk of transmission of the virus, and to allocate equipment, hospital beds, and manpower to combat COVID-19^11^ ^-^ ^12^. In inguinal hernia, many patients can be safely followed up with a very low probability of developing complications, such as incarceration and the need for urgent surgery, situations which predispose to greater morbidity and mortality^5^. However, the increase in the occurrence of emergency repairs with the follow-up strategy reinforces the indication of surgery for most patients if clinical conditions allow^5^.

With the need to interrupt elective procedures due to the spread of SARS-CoV-2, we observed that the number of hospitalizations for unilateral inguinal hernioplasty in Brazil during the peak period of the pandemic in the country decreased significantly, 55.21%, when compared with the previous year.

Our findings are consistent with those previously reported in the literature, suggesting that the impact of COVID-19 on surgical practice has affected the entire world. The literature reports a 40-86% decrease in non-traumatic surgical emergencies after the virus outbreak^13^ ^-^ ^16^.

Due to the pause in elective surgeries, many patients possibly evolved with complications of their conditions^9^. In the case of inguinal hernia, incarceration and/or ischemia of the herniated contents may occur, leading to serious complications, such as systemic infections, intestinal obstruction, and intestinal tissue necrosis. In this context, the repair becomes an emergency surgery, and a procedure with low complexity and mortality becomes riskier^17^. A recent meta-analysis showed that an elective hernia repair has a mortality rate of 0.2%, while in the emergency context it reaches 4%^17^.

In this study, we found an increase in the mortality rate of 81.81% when compared with the year before the COVID-19 pandemic, despite the large reduction in the number of hospitalizations for this type of surgery.

April 2020, displayed the peak in mortality. The Brazilian average in that month was 0.47, a significant increase compared with the value of 0.05 in the same month in the previous year. The highest value observed was in the Northeast region, where in April 2019 the mortality rate was 0.17, reaching 1.27 in April 2020. The North region recorded a mortality rate of 0.58 in that month. The other regions did not record mortality data for this period in DATASUS.

This significant increase in mortality can be explained by the patient’s infection with COVID-19 and the fact that surgeries were then performed only on an emergency basis. Hernia repair is a surgical procedure with low morbidity and mortality, but in patients infected with COVID-19 it can be fatal^11^. Depending on the severity of an epidemic and the availability of resources, the risks and benefits of elective surgical procedures must be carefully evaluated. In addition, due to the pause in elective surgeries, many patients possibly evolved with complications of the condition^9^.

In a study carried out at a University Hospital in Florence^18^, Italy, which compared the number of surgical procedures in March 2019 with March 2020, hernia repair was reduced by 48%. No statistically significant differences were found regarding age, sex, mortality, postoperative ICU stay, and time between admission and surgery. However, mortality in patients with COVID-19 was 25% versus 7% in patients not infected by the SARS-CoV-2^18^.

A retrospective analysis performed at a Hospital in Porto Alegre, Southern Brazil^19^, compared patients undergoing emergency surgery for acute appendicitis during the months of March and April 2020, with the same months of the previous year. The number of appendectomies during the pandemic dropped by 56%, and the median time from symptom onset to hospital arrival was significantly longer in 2020 (40.6 vs. 28.2 hours, p=0.02). They also observed a significantly higher proportion of complicated cases than in the previous year (33.3% vs. 15.2%, p=0.04)^19^.

As for the average hospital stay, we observed an increase in relation to the previous year. In April 2020, there was an increase in the average hospital stay in all regions. The highest rate was 2.7 in the South, which in April of the previous year was 1.3. As the number of hospitalizations decreased, a possible explanation for these data is that patients are more susceptible to the transmission of the virus, inside and outside the hospital environment, and thus, prolonging hospitalization due to infection by the SARS-CoV-2. Another factor that may have impacted the hospitalization period is the restriction to emergency surgical situations, which are more severe and have greater risk of complications. In this context, the prevalence of this type of surgery may also have contributed to the increase in the rate of hospital stay.

It is worth mentioning that the SIH/SUS does not have data on age, comorbidities, hernia severity, complications, and COVID-19 infection of hospitalized patients. This limitation prevents a more detailed assessment of the causes of the procedure increased mortality during this period, since these data impact patients’ prognosis and outcome.

With the decrease in COVID-19 cases, there was a return to non-emergency activities in some hospitals. Since July 2020, there has been an increase in the number of hospitalizations in all regions, reaching its highest value in November 2020, still with much lower values than before the arrival of the SARS-CoV-2 virus in Brazil. However, in December 2020 there was again a decline, due to the new increase in the incidence of COVID-19 cases in the country, and the need to return to restrictions.

The long-term effects of the accumulation of surgeries and the impact of the COVID-19 pandemic on patient survival will still take some time to be clarified. A major effort will be needed to reduce outstanding surgical cases. In this period of uncertainty, it is necessary to use the best scientific evidence, adapt protocols, and develop a service recovery plan, prioritizing patients with clinical conditions with greater risk of deterioration^20^.

## CONCLUSION

The COVID-19 pandemic has significantly altered the total number of surgeries performed and mortality rates, especially during the peak of the pandemic in the country. We observed that the number of hospitalizations for unilateral inguinal hernioplasty in the period of the COVID-19 pandemic in Brazil was reduced by 55.21% compared with the previous year. However, there was a significant increase in the procedure mortality rate. Hernia repair has low morbidity and mortality, but in patients infected with COVID-19, it can be fatal. In addition, the restriction to performing only emergency surgeries during the peak period of the pandemic in the country may have significantly influenced these results, as they consist of more complicated conditions that lead to higher mortality.
